# How Social Exclusion Affects Consumers’ Color Preference

**DOI:** 10.3389/fpsyg.2022.850086

**Published:** 2022-08-03

**Authors:** Lu Zong, Shali Wu, Shen Duan

**Affiliations:** ^1^School of Management, Kyung Hee University, Seoul, South Korea; ^2^School of Business, Renmin University of China, Beijing, China

**Keywords:** social exclusion, consumer preference, warm color, cold color, self-threat, self-construal

## Abstract

Social exclusion can cause negative changes on human beings both in the physiological and psychological aspects. Although considerable efforts have been devoted to study its effects on consumption behavior, little attention has been paid to the consequence that social exclusion might have on consumer’s color preference and the underlying mechanisms. Such social events can change individual’s behavior. This work examines the influence of social exclusion on consumers’ color preference as well as the moderation and mediation effects *via* three experiments: Experiment 1 studies the impacts of social exclusion on consumer color choice (warm color versus cold color). To further validate the robustness of the results, experiment 2 is designed by replicating the findings of experiment 1 in another product category and instructed the participants to choose products with different colors. Meanwhile, the mediation effect of self-threat is examined. In Experiment 3, the moderation effect of self-construal is investigated *via* a 2 (exclusion vs. inclusion) × 2 (independent vs. interdependent) × (warm color vs. cold color) between-subjects design. Our results indicate that social exclusion makes people prefer warm colors rather than cold colors. However, these effects would be mediated by self-threat, which could be further moderated by self-construal. The present study establishes the relationship between social exclusion and consumers’ color preference, which is expected to provide guidance for companies to improve product design and promotion strategies to adapt to various contexts.

## Introduction

Social exclusion refers to negative interpersonal experience in which an individual or a group is excluded or rejected by others. In daily life, human beings always suffer from various kinds of social exclusion, such as being neglected, discriminated, abused, or bullied, etc. As one of the most common social experience, social exclusion exists everywhere (Williams, 2007; Nezlek et al., 2015). For example, friend requests are declined by others *via* social platforms; membership application into a certain club is denied; credit card application is rejected by banks. It is reported that nearly 80% of people had been excluded at their workplace (O’Reilly et al., 2015).

In recent years, increasing evidence suggested that social exclusion can change individual’s behaviors, leading to diverse consumption patterns like conformity consumption (Mead et al., 2011), conspicuous consumption (Lee and Shrum, 2012), luxuries consumption (Molden et al., 2009; Lee and Shrum, 2012), uniqueness and status consumption (Wan et al., 2014). For instance, social excluded individuals are likely to feel their social status in existing relationships is diminished or lost, thus they tend to seek social acceptance through conspicuous consumption (Molden et al., 2009; Lee and Shrum, 2012). Studies also revealed social excluded customers prefer to buying products of nostalgia (Loveland et al., 2010), anthropomorphism (Chen et al., 2017), for consuming such products helps to make up for their internal defects and alleviate the negative effects of social exclusion. Despite a series of research has been performed to study the consumption types and their effects on the elimination of social exclusions, it is still unclear whether the appearance of products would impact the negative impacts of social exclusion or not, especially regarding the color of the products.

Color is an essential element that plays important roles in sensory consumption. Indeed, it has long been recognized as one of the most representative visual cues concerning the possible sensory properties. To date, multiple laboratory research has demonstrated that altering the product color can exert a dramatic influence on the expectations, and hence on the subsequent experiences of consumers (Spence, 2015b). As a consequence, an emerging research field lies in the sensory marketing in terms of color, which considerably influences consumer decision making. A series of questions naturally arise: does social exclusion affect people’s choice for products of different colors? If so, what colors do they prefer? What are the underlying psychological mechanisms? Although remarkable progress has been achieved both in the social exclusion and sensory marketing field, there is still rather limited research focusing on the impact of social exclusion on consumers’ product preferences from a sensory marketing perspective. Little attention was paid from the sensory marketing perspective to explicit how social exclusion influences consumers’ color preference.

Based on the compensatory consumption theory, this study systematically discusses the above problems by applying the empirical method. Hopefully it can correlate social exclusion and sensory marketing together, and to further clarify the underlying mechanisms.

## Social Exclusion

Social exclusion, including social rejection, social exclusion, and ostracism, has been widely discussed across multi-disciplines, such as social psychology (Doolaard et al., 2020), anthropology (Peruzzi, 2014). People often experience instances of being ignored or rejected in their relationships with family, friends, colleagues, or acquaintances (Rajchert and Winiewski, 2017), making them feel they are excluded from others, and cannot integrate into the group. Thus, they cannot acquire the sense of belonging of the group. In addition, social exclusion causes a series of negative effects on people’s cognition, physiology and psychology. For example, social exclusion increases aggression and decreases helping behavior toward the excluder, while encourages more prosocial behavior to new potential relationship partners (Rajchert and Winiewski, 2017). Due to social exclusion, the perception of time can be distorted (Bargh and Shalev, 2012; IJzerman et al., 2012). Furthermore, it directly triggers negative psychological reactions in the excluded person, such as anxiety, loneliness, jealousy, depression, and other negative emotions (Freedman et al., 2016).

In recent years, the theoretical concept of social exclusion has been applied to consumer behavior by scholars, primarily focusing on the responses of the social-excluded consumers in the aspects of their cognitive, physiological, psychological, and purchases behaviors. When applied to the field of the consumer behavior, it is seen that people will compensate the negative feeling of social exclusion through consumption to regain their social inclusion. Thus, the choice of product is a strategic behavior which orients toward the goal of gaining a sense of social belonging. The impact of social exclusion on consumer behavior has been investigated by many marketing scholars from the perspective of relationship facilitation responses. In response to the negative experience of social exclusion, consumers may develop a preference for products of particular characteristics. Specifically, to gain a sense of belonging and to repair social relationships, social excluded consumers are more inclined to purchase products related to group identity, but hide their real consumption habits instead of imitating the consumption preferences of social objects. This study explains their preference for warm and cool colors from this perspective.

## Social Exclusion and Self-Threat

Social exclusion raises several issues that trigger self-threat (influenced by an individual’s trait self-esteem). Belongingness is one of the fundamental needs of human beings, but can be threaten by social exclusion. As a result, it brings a series of negative consequences, like severe psychological, behavioral, and cognitive impairments. A salient example was reported by Eck et al. (2017), in which social exclusion was found to threaten the basic needs of human, including belonging, self-esteem, control, and meaningful existence. As revealed by Stillman et al. (2009), social exclusion would make individuals perceive life as less meaningful. It reminds people of the fragility of their lives and is therefore often perceived as social death (Williams et al., 2000), leading to negative reactions such as depression (Baumeister and Tice, 1990), anger, sadness (Williams et al., 2000). In addition to self-threat, social exclusion causes decrements in self-regulation, further reduces the sense of self-control. Excluded individuals tend to strive for a positive self-view and use all kinds of strategies to reestablish or heighten their self-esteem (Hoefler et al., 2015).

In summary, social exclusion can lead to self-threat in various domains like self-regulation, self-control, and even individual’s existence. As a fundamental interpersonal relationship, attribution needs to be re-associated with others when it is threatened. The self-threat caused by the threats in these fields may have an impact on individuals’ subsequent behaviors. Consumers in the experience of social exclusion will purchase specific products through specific consumption decisions in response to the psychological needs. This study focuses on how the social exclusion influences the consumption decisions and their subsequent behaviors when they experience rejection and feel self-threatened.

## Color and Self-Threat

The choice for products can partially reflect self-characteristic, and have symbolic value to manifest and repair the self. Thus, consumer behavior closely related to self-threat (Mead et al., 2011; Koles et al., 2018; Wang et al., 2022). Previous research has shown that consumers who experienced self-threat from social exclusion will adopt appropriate consumption behaviors to alleviate this self-threat. For example, individuals lacking a sense of belonging may choose nostalgic products (Loveland et al., 2010), warm or comfort food (Troisi and Gabriel, 2011; Wang et al., 2020), luxury or anthropomorphic products (Lee and Shrum, 2012; Mourey et al., 2017), by evoking their sense of contact with the same consumer groups to meet the needs of attribution. By expanding the subject further into the visual appearance of products, this study tries to explore whether the color of products are also effective in reducing self-threat related negative effects.

As one of the most important elements in vision, color is ubiquitous and has specific meanings. With technology advancement and the reduction of manufacturing cost, consumers can choose different colors to match and express their personality. Marketers often use color in advertising, product design, packaging design, brand design, and in-store environment to improve consumers’ evaluation and purchase willingness. The earliest study on color and psychology appeared in 1931 (Gunlach and Macoubrey, 1931). Subsequently, abundant researches were carried out to investigate the relationship between color and consumer behavior as well as the underlying mechanisms. The past decade has witnessed increased interest in research on color and psychological functioning (Babin et al., 2003; Meyers-Levy and Zhu, 2007; Hagtvedt and Brasel, 2016). Color has been applied as an important marketing tool used by businesses, which not only conveys a meaning or message in advertising, but also is capable of getting the attention of individuals. For instance, by using color schemes in stores that don’t clash or overwhelm customers, it is possible to increase the probability of the potential customer purchasing. Color advertising can influence consumers to pay more for products with unnecessary extras, whereas black-and-white advertising gets them to focus on basic product features (Puccinelli et al., 2013).

Interestingly, humans perceive colors as warm and cold regardless of actual temperature, which is related to the dominant wavelength of the color. Colors with longer wavelengths are warm colors (e.g., red, yellow, orange), while those with shorter wavelengths are described as cold (e.g., blue, green, purple). It is reported that color is able to trigger emotional responses to the stimuli in a more subliminal way than text or images. In general, warm colors are regarded as active and stimulating (Valdez and Mehrabian, 1994). Specifically, red is regarded as the warmest color, symbolizing the sun and fire, which creates psychological warmth. It will cause a higher level of awakening and improve attractiveness and competitive performance (Elliot and Maier, 2012). In contrast, blue is considered to be the coldest color, which creates psychological coldness and makes consumers feel more relaxed and calm (Labrecque and Milne, 2012; Elliot and Maier, 2014). In offline environment, cold-color background can decrease the likelihood of postponing purchase compared to warm-color background. Thus, violet/blue interiors will lead to increased purchase intentions than will red/orange interiors. In the auction situation, red background tends to make consumers offer higher price than blue background. However, in the bargaining situation, the red background will make consumers bid lower (Bagchi and Cheema, 2013).

Individuals who experience social exclusion have negative psychological feeling (Williams, 2007), which in turn, drives them to select things that can eliminate their negative psychological experiences. In specific product choices, warm colors create a psychological feeling of warmth, and can compensate for the negative psychological experience of social exclusion. It is reported that warm colors can eliminate negative psychological experiences such as loneliness, and bring positive feelings such as warmth, and pleasure (Elliot and Maier, 2014). In order to eliminate the negative psychological experiences of social exclusion, lowered sense of belongingness, and reduced self-esteem, the excluded would prefer warm colors rather to cold colors. Based on the above analysis, the following hypotheses are proposed.

H1: Social exclusion affects consumers’ preferences of cold and warm colors. Specifically, compared with socially included consumers, excluded consumers are more likely to choose warm color products.

H2: Self-threat mediates the effect of social exclusion on consumers’ preference for warm color (vs. cold) products.

## The Moderating Role of Self-Construal

Self-construal is conceptualized as a “constellation of thoughts, feelings, and actions concerning one’s relationship to others, and self as distinct from others” (Pilarska, 2014). Briefly, it is a way for individuals to recognize and perceive themselves. In general, self-construal can be divided into interdependent self-construal and independent self-construal. Under the stimulation of a certain situation, independent self-constructed individuals tend to exhibit self-centered behavior. However, interdependent self-constructed individuals exhibit more “other-centered” behavior. For them, social identity is particularly important (Trafimow et al., 1991). Maintaining the integrity and closeness of their social network is one of the starting points for their behaviors in daily life. Therefore, interdependent self-constructed individuals are more sensitive in social situations, and prefer to be perceived to be more generous and considerate by others (Lalwani and Shavitt, 2009). As a result, they are more likely to expect harmonious interpersonal relationships, and will be more likely to choose social relationship compensatory products.

The goal of the interdependent selves is to gain social acceptance and maintain one’s social image. Thus, they often take into account the perceptions of others and society when making decisions. On the contrary, the goal of the independent selves is to establish their own uniqueness and differentiation from others, to focus on their own thinking, and to follow their own hearts (Hong and Chang, 2015). They have been accustomed to living or behaving in their own manner, and don’t perceive social harmony as an important element in their life. In other words, they tend to feel it doesn’t matter of being social excluded, for it only means a temporary disconnection of social links. For individuals of interdependence, they feel self-threat by social exclusion, since they desire to be connected to society. Therefore, the following hypotheses are proposed:

H3: Self-construal plays a moderator role in main effect. Specifically, compared to independent self-construal, interdependent self-construal will express greater preference for warm colors.

## Overview of Studies

**FIGURE 1:**
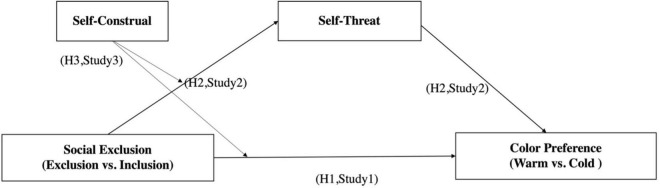
Conceptual framework.

In this work, three experiments have been performed to test the proposed hypotheses (see Figure 1). Study 1 is a between-subjects design, aiming to test the basic predictions (H1). Study 2 demonstrates the processing mechanism of these relationships. Finally, Study 3 replicates the findings of Study 2, and shows an important boundary condition of the effect of social exclusion (i.e., interdependent vs. independent). To demonstrate the versatility of findings, we manipulate warm and cold colors using different products (i.e., cellphone, car), and adopt three types of social exclusion scenarios.

## Study 1

### Design and Procedure

The purpose of Study 1 was to test our basic assumption by employing 2 × 2 (state of social exclusion: exclusion vs. inclusion) × (warm and cold color product: red vs. blue) between-subjects design. Since blue and red are on opposite sides of the color spectrum and regarded as the coldest and warmest color respectively (Ballast, 2002), they were firstly studied. A total of 240 (101 males and 139 females, M_age_ = 29.83, SD = 7.04) adults participated in this experiment for monetary compensation and were asked to complete several unrelated tasks. Participants were randomly assigned to 2 × 2 conditions (state of social exclusion: exclusion vs. inclusion) × (warm and cold color product: red vs. blue) between-subjects design. The experiment consisted of two seemingly independent tasks. The first task was a situational imagery investigation aiming to manipulate individuals of social exclusion and social inclusion, adapting to the experiments by Wan et al. (2014). Specifically, participants were asked to read a story and imagine themselves in the situation: “IWE is an international gaming company, and members who successfully apply for membership in the IWE House are entitled to a range of exclusive value-added services. You are interested in joining the IWE Member House and have sent out your application.” We then manipulated the two types of social exclusion with different responses, for the rejection group: after 1 week, you receive a rejection letter from the IWE Member House, and for the social inclusion group: after 1 week, you find that you are warmly welcomed by the IWE Member House. Next, the subjects were asked to record in detail the scene they had just recalled, including what happened and how they felt at the time. The manipulation test scale of Molden et al. (2009) was used to measure the extent to which subjects felt rejected and ignored, and the two items of the scale were “To what extent did you feel rejected in the scene you just recalled? And “To what extent did you feel ignored in the scene you just recalled? (1 = not at all, 7 = very strongly, *r* = 0.92).

The second experimental task was a product attitude survey. Participants were told that they were going to purchase a car for themselves, and were asked to indicate their choice of the product promoted by either a warm color cue (this car is red) or a cold color cue (this car is blue). The two cars are completely the same except the color. To minimize the possibility of being influenced by the brand impression, the logo and other identifiable elements were blurred before tests.

Participants then reported their purchase intention on a seven-point scale anchored by 1 = not at all, 7 = very much. The purchase intention scale is based on the “purchase intention scale” used by Dodds et al. (1991) (e.g., “I have a very high intention to buy this product” and “The likelihood of purchasing this product is very high.”) (1 = not at all, 7 = very strongly, r = 0.77), then reported on the mood (Williams et al., 2000), product distinctiveness (Rego et al., 2009), demographic variables, before ending the experiment.

### Results and Discussion

#### Manipulation Check

Two measuring modes of social exclusion were used, which were “being ignored” and “being rejected”. The perception difference in the social exclusion of participants under two different measuring modes were examined, together with the average of the two modes in the control test. The results showed that subjects in the social exclusion group had a stronger sense of being ignored than those in the social acceptance group [M_social exclusion–sense of being ignored_ = 5.59, SD = 1.36 vs. M_social acceptance–sense of being ignored_ = 2.13, SD = 1.53; *F*_(1,238)_ = 343.74, *P* < 0.001]; Similarly, participants in the social exclusion group had a stronger sense of being rejected than those in the social acceptance group [M_social exclusion–ssense of being rejected_ = 5.23, SD = 1.52 vs. M_social acceptance–sense of being rejected_ = 1.90, SD = 1.45; *F*_(1,238)_ = 301.45, *P* < 0.001]. Then, after averaging the score of being ignored and rejected of the subjects, the perceptual difference of the subjects under different degrees of social exclusion was investigated. The results have shown that compared to participants in the social inclusion group, participants in the social exclusion group felt a stronger sense of social exclusion [M_social exclusion_ = 5.41, SD = 1.27 vs. M_social inclusion_ = 2.02, SD = 1.41; *F*_(1,238)_ = 384.30, *p* < 0.001].

##### Color Perception

Using a one-way ANOVA test, it was found that participants reckoned that the color of red cars (M_red_ = 6.02, SD = 0.97) was warmer than that of blue cars [M_blue_ = 3.03, SD = 1.63; *F*_(1,238)_ = 297.99, *P* < 0.001], proving our control of the color was successful.

##### Confounding Check

A two-way ANOVA was carried out using social exclusion and product color as independent variables and subjects’ emotions as dependent variables. The results have shown that the main effect of social exclusion was significant [*F*_(1,236)_ = 101.82, *p* < 0.001, η^2^ = 0.30], whereas the main effect of product color [*F*_(1,236)_ = 0.17, *p* = 0.682], and the interaction effect between social exclusion and product color was not significant [*F*_(1,236)_ = 0.97, *p* = 0.327]. This indicates that the degree of social exclusion would influence the emotion difference. Therefore, emotion was used as a concomitant variable for control in the subsequent analysis. Similarly, a two-way ANOVA was carried out with social exclusion and product color as independent variables and product uniqueness as a dependent variable. The results have shown that the main effect of social exclusion was significant [*F*_(1,236)_ = 9.18, *p* = 0.003, η^2^ = 0.04], the main effect of product color was not significant [*F*_(1,236)_ = 1.65, *P* = 0.201], while the interaction effect between social exclusion and product color was significant [*F*_(1,236)_ = 4.88, *p* = 0.028, η^2^ = 0.02], suggesting that the degree of social exclusion would affect the uniqueness judgment on vehicles of different colors. Therefore, the product distinctiveness was used as a concomitant variable for control in the subsequent analysis.

##### Product Purchase Intention

A multi-factor covariance analysis was carried out with social exclusion and product color as independent variables, emotion and product uniqueness as concomitant variables, and product purchase intention as a dependent variable. The results have shown that after controlling the participants’ emotion and product distinctiveness perception, the main effect of social exclusion was not significant [*F*_(1,234)_ = 0.23, *p* = 0.631], and the main effect margin of product color was significant [*F*_(1,234)_ = 3.71, *P* = 0.055, η^2^ = 0.02], while the interaction effect between social exclusion and product color was significant [*F*_(1,234)_ = 13.51, *p* < 0.001, η^2^ = 0.06]. Through a further analysis of planned contrast, it was found that subjects in the social exclusion group showed significantly higher intention to buy red (warm color) cars than blue (cool color) cars [M_social exclusion–red purchase_ = 5.46, SD = 1.32 vs. M_social exclusion–blue purchase_ = 4.30, SD = 1.71; *F*_(1,236)_ = 21.64, *p* < 0.001, η^2^ = 0.08]; On the contrary, there was no significant difference between the purchase intention of red (warm color) and blue cars (cool color) in the social inclusion group [M_social inclusion–red purchase_ = 5.29, SD = 1.33 vs. M_social inclusion–blue purchase_ = 5.65, SD = 1.01; *F*_(1,236)_ = 2.07, *p* = 0.151]. Therefore, Hypothesis 1 has been preliminarily verified.

## Study 2

By extending the findings of Study 1, Study 2 aims to further investigate the psychological mechanism underlying the social exclusion effect by testing the mediating effect of self-threat, as well as ruling out potential confounds (such as product distinctiveness, emotion).

### Design and Procedure

In Experiment 2, a total of 240 subjects were recruited on the Credamo platform. The experiment was carried out using a two-factor intergroup design of 2 (state of social exclusion: exclusion vs. inclusion) × 2 (Product color: warm color vs. cool color). There were 102 male subjects (42.50%) and 138 female subjects (57.50%), with an average age of 28.77 years (SD = 5.67). The experiment consisted of two seemingly independent tasks. The first task was a situational imagery investigation, aiming to manipulate social exclusion, a method adapted from Lu and Sinha (2017) manipulation of social exclusion and inclusion. Specifically, participants were asked to read a story and imagine themselves in the situation below:

This is the beginning of the new semester, and you do not know many people in your class. You are taking a marketing class in which you must work on multiple assignments in groups. You must find three students to form a group. After a couple of classes, you decide to ask three students, because you have some conversations with these three students during/after the class and they are seemingly friendly. You then send an email request to each of these three students and ask whether they would like to work together with you for the group assignments. A day later, you receive emails from them, and all students reject (accept) your requests to work in a group together.

After reading this scenario, participants were asked to indicate how rejected and ignored they felt during the experience. The manipulation test scale of Molden et al. (2009) was used to measure the extent to which subjects felt rejected and ignored, and the two items of the scale were “To what extent did you feel rejected in the scene you just recalled?” and “To what extent did you feel ignored in the scene you just recalled?” (1 = not at all, 7 = very strongly, *r* = 0.92).

After completing the social exclusion manipulation, participants were asked to indicate their perceived self-threat using nine items adapted from Baumeister (2002) and Campbell et al. (2003) (“In such a situation, I would try harder to restrain my bad emotions”; There will be an inexplicable depression deep inside yourself; The presence of such a service situation can make me feel irritable or uneasy inside; This scenario experience makes me feel a kind of hard to say; It will prevent me from communicating with my companion; I would feel very humiliated if such a situation arose; I feel disrespected; In this situation, I was able to present myself better; This is not conducive to my social interaction”) (1 = not at all, 7 = very strongly).

In the final section of the experiment, participants were told that they were going to purchase a phone for themselves from an online retailer. They were asked to indicate their choice of the product promoted by either a warm color cue (this cellphone is red) or a cold color cue (this cellphone is blue). The two phones are completely same equipped except the color. To minimize the possibility of being influenced by the brand impressions, the logo and other identifiable elements were blurred before tests.

Participants then reported their purchase intention on a seven-point scale anchored by 1 = not at all and 7 = very much. The purchase intention scale is based on the “purchase intention scale” used by Dodds et al. (1991) (e.g., “I have a very high intention to buy this product” and “The likelihood of purchasing this product is very high.”) (1 = not at all, 7 = very strongly, r = 0.77), then report on the mood (Williams et al., 2000), product distinctiveness (Rego et al., 2009), and demographic variables, before ending the experiment.

### Results and Discussion

#### Manipulation Check

Two measuring modes of social exclusion were used, which were “being ignored” and “being rejected.” The perception difference in the social exclusion of participants under two different measuring modes were examined, along with the average of the two modes in the control inspection. The results showed that participants in the social exclusion group had a stronger sense of being ignored than those in the social acceptance group [M_social exclusion–sense of being ignored_ = 5.60, SD = 1.31 vs. M_social acceptan*ce*–sense of being ignored_ = 1.95, SD = 1.26; *F*_(1,238)_ = 482.24, *P* < 0.001]; Similarly, participants in the social exclusion group had a stronger sense of being rejected than those in the social acceptance group [M_social exclusion–sense of being rejected_ = 6.31, SD = 1.15 vs. M_social acceptance–sense of being rejected_ = 1.72, SD = 1.38; *F*_(1,238)_ = 784.12, *P* < 0.001]. Then, after averaging the score of being ignored and rejected of the subjects, the perceptual difference of the participants under different degrees of social exclusion was investigated. The results showed that compared to subjects in the social inclusion group, subjects in the social exclusion group felt a stronger sense of social exclusion [M_social exclusion_ = 5.95, SD = 1.09 vs. M_social inclusion_ = 1.83, SD = 1.27; *F*_(1,238)_ = 728.94, *p* < 0.001], implying the successful manipulation of social exclusion.

##### Color Perception

Using a one-way ANOVA, it was found that subjects reckoned that the red cellphone (M_red cellphone_ = 6.07, SD = 1.00) was warmer than the blue cellphone [M_blue cellphone_ = 2.89, SD = 1.55; *F*_(1,238)_ = 355.38, *p* < 0.001], proving that our control of the color was successful.

##### Confounding Check

An ANOVA was carried out using social exclusion as an independent variable and subjects’ emotions as dependent variables. The results showed that the degree of emotional happiness of the subjects in the social acceptance group (M_social acceptance_ = 5.66, SD = 1.21) was significantly higher than that in the social exclusion group [M_social exclusion_ = 3.36, SD = 1.52; *F*_(1,238)_ = 166.91, *p* < 0.001]. It could be seen that there was significant difference in the emotions of different social exclusion groups. Therefore, subjects’ emotion was used as a concomitant variable in the subsequent analysis to control its influence on the results of the study. Secondly, color control was selected as an independent variable and variance test using subjects’ perception of cellphone uniqueness as a dependent variable, the results have shown that the main effect of social exclusion was not significant [*F*_(1,238)_ = 1.88, *p* = 0.172], which had effectively excluded the confounding effect of product distinctiveness. Therefore, the confounding effect caused by product distinctiveness was not considered in the subsequent analysis.

##### The Main Effect Test of Self-Threat

**FIGURE 2:**
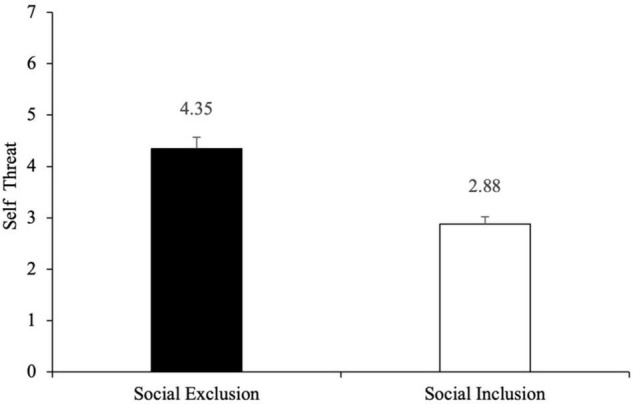
Effects of social exclusion vs. social inclusion on self-threat. Error bars represent standard errors.

A multi-factor analysis of covariance was carried out using social exclusion and product color as independent variables and self-threat as dependent variables. The results have shown that the main effect of social exclusion was significant [*F*_(1,236)_ = 127.49, *p* < 0.001, η^2^ = 0.35], while the main effect of color control [*F*_(1,236)_ = 0.03, *p* = 0.864] and the interaction effect between social exclusion and color control was not significant [*F*_(1,236)_ = 0.09, *p* = 0.765]. This reveals that only the difference in social exclusion would influence individual’s self-threat, while color control had no obvious influence on self-control. As expected, see Figure 2, subjects in the social exclusion group felt a stronger sense of self-threat than those in the social inclusion group [M_social exclusion–sense of self–threat_ = 4.35, SD = 1.19 vs. M_socialinclusion–sense of self–threat_ = 2.88, SD = 0.77; *F*_(1,238)_ = 128.51, *p* < 0.001].

#### Product Purchase Intention

**FIGURE 3:**
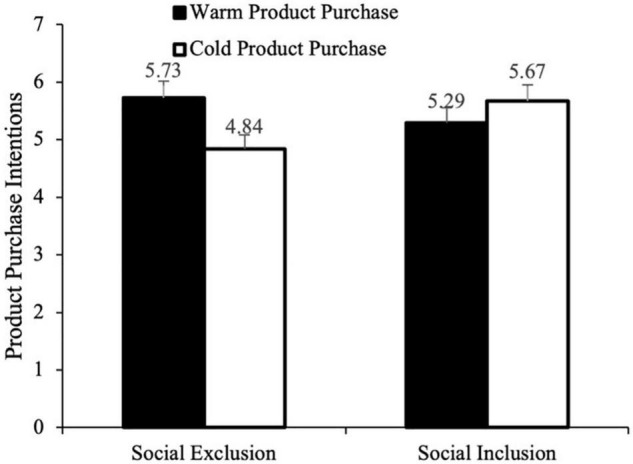
The interaction of social exclusion and warm and cold color’s type (Study 2).

For the main effect test of product purchase intention, a multi-factor covariance analysis was carried out with social exclusion and product color as independent variables, emotion as a concomitant variable, and product purchase intention as a dependent variable. The results have shown that after controlling the subjects’ emotions, the main effect of social exclusion [*F*_(1,235)_ = 6.00, *p* = 0.015, η^2^ = 0.03] and the main effect margin of product color was significant [*F*_(1,235)_ = 3.25, *p* = 0.073, η^2^ = 0.01]. More importantly, the interaction effect between social exclusion and product color was significant [*F*_(1,235)_ = 19.08, *p* < 0.001, η^2^ = 0.08]. The results of further analysis of planned contrast have shown that participants in the social exclusion group showed much higher intention to buy red (warm color) cellphones than blue (cool color) cellphones [M_social exclusion–purchase of red cellphone_ = 5.73, SD = 0.85 vs. M_social exclusion–purchase of blue cellphone_ = 4.84, SD = 1.80; *F*_(1,236)_ = 14.33, *p* < 0.001, η^2^ = 0.06]; On the contrary, no significant difference between the purchase intention of red and blue cellphones is observed in the social inclusion group [M_social inclusion–purchase of red cellphone_ = 5.67, SD = 0.90 vs. M_social inclusion–purchase of blue cellphone_ = 5.29, SD = 1.33; *F*_(1,236)_ = 2.58, *p* = 0.109], see Figure 3. Hypothesis 1 has been verified.

#### Mediation Analysis

**FIGURE 4:**
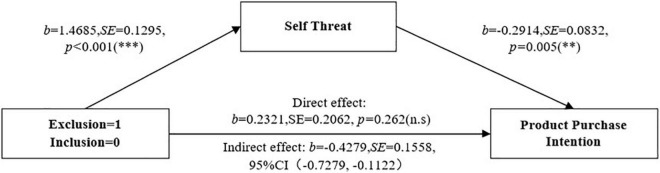
Study 2: the mediation effect of self-threat. ^∗^*p* < 0.05, ^∗∗^*p* < 0.01, ^∗∗∗^*p* < 0.001. *Significance at the 0.05 level.

An analysis of the mediating effect of self-threat was carried out according to the Bootstrap method of Hayes (2013), in which Model 4 in the Process was used for the mediation test. The sample size was 5,000. Social exclusion was used as an independent variable (exclusion = 1, inclusion = 0), self-threat was used as an intermediary variable, and product purchase intention was used as a dependent variable. The results have shown that at the 95% confidence interval, self-threat had a significant indirect mediating effect on product purchase intention in the social exclusion group (LLCI = –0.7279, ULCI = –0.1122). The estimated value of mediating effect was –0.43. Moreover, after self-threat was controlled, the direct effect of social exclusion on product purchase intention was not significant [LLCI = –0.1742, ULCI = 0.6384, see Figure 4], suggesting that self-threat played a whole mediating role in the relationship between social exclusion and purchase intention of warm color products, thus Hypothesis 2 was verified.

## Study 3

To further clarify the underlying process, Study 3 investigates the moderating role of self-construal. Besides, to further validate the robustness of our findings, we manipulated social exclusion in a marketing context, where product stimuli was changed into pictures of Airbnb apartments.

### Design and Procedure

In Experiment 3, a total of 500 subjects were recruited on the Credamo platform, including 195 male (39.00%) and 305 female (61.00%), with an average age of 28.61 years (SD = 5.11). Participants were randomly assigned to two by two conditions (state of social exclusion: exclusion vs. inclusion) × (warm & cold color product: orange vs. purple) between-subjects design, and then were asked to complete several unrelated tasks. Following same procedure as Study 1, Social exclusion was manipulated using a virtual marketing service scenario, a method adapted from Ward and Dahl (2014) manipulation of social exclusion and inclusion. The participants first read a story and put themselves in the role of the main character, which involved their dining experience at a restaurant. In the social inclusion group, the participants were treated with hospitality and attention by the waiter, while the social exclusion group was ignored by the waiter during the meal. After completing the task, we asked participants to indicate the extent to which they felt rejected (accepted) on a seven-point scale for a manipulation check (1 = not at all, 7 = very much) (Lu and Sinha, 2017).

#### Social Inclusion

Imagine that you are dining indoors at a restaurant. As you enter the restaurant, you received warm hospitality and attention from the waiters in the restaurant. They warmly greet you and (condescendingly) show where your seat is and ask if they can help you with your order. You point the menu and asked them what’s the ingredients of the food, and they reply you patiently.

#### Social Exclusion

Imagine that you are dining indoors at a restaurant. As you enter the restaurant, there are no waiters from the restaurant to welcome you. You need to find available seat by yourself, the waiters just ignore you (even they are available). When you plan to order your food from the menu, and want to know the ingredients of the food, there are no waiters paying attention to you.

After completing the social exclusion manipulation, participants were asked to indicate their self-construal, which was measured using a 14-item scale developed and validated by Ma et al. (2014). Sample items for interdependence (seven items; α = 0.78) include “Parents and children must stay together as much as possible” and “I feel good when I cooperate with others.” Sample items for independence (seven items; α = 0.69) include “I’d rather depend on myself than others” and “I often do “my own thing.””

Subsequently, participants were instructed to imagine themselves in a purchase scenario and were told that they were planning to travel and would like to rent an apartment on Airbnb, a service website that connects travelers with homeowners who have rooms available for rent and provides users with a wide variety of accommodation information. The two apartments are completely same design, and the only difference is the color. This product selection scenario was adapted from Torres et al. (2020). They were asked to indicate their choice of the product promoted by both a warm color cue (orange) and a cold color cue (purple). Participants then reported their purchase intention on a seven-point scale anchored by 1 = not at all and 7 = very much. Participants were then asked to measure their self-threat. Similar to Study 2, items of self- threat adapted from Baumeister (2002) and Campbell et al. (2003).

In the final section of the experiment, Participants were evaluated the color of the orange apartment and the purple apartment (1 = totally cool, 7 = totally warm). Participants filled out the mood (Williams et al., 2000), product distinctiveness (Rego et al., 2009). Finally, they provided brief demographic information and were thanked for their participation.

### Results and Discussion

#### Manipulation Check

Since two measuring modes of social exclusion were used, which were “being ignored” and “being rejected,” therefore, the perception difference in the social exclusion of subjects under two different measuring modes as well as the average of the two modes were examined in the control test. The results showed participants in the social exclusion group had a stronger sense of being ignored than those in the social acceptance group [M_social exclusion–sense of being ignored_ = 6.06, SD = 1.30 vs. M_social acceptance–sense of being ignored_ = 1.96, SD = 1.39; *F*_(1,498)_ = 1161.64, *p* < 0.001]; Similarly, participants in the social exclusion group had a stronger sense of being rejected than those in the social acceptance group [M_social exclusion–sense of being rejected_ = 5.48, SD = 1.46 vs. M_social acceptance– sense of being rejected_ = 1.81, SD = 1.36; *F*_(1,498)_ = 843.70, *p* < 0.001]. Then, after averaging the score of being ignored and rejected of the subjects, the perceptual difference of the subjects under different degrees of social exclusion was investigated. The results have shown that compared to participants in the social inclusion group, subjects in the social exclusion group felt a stronger sense of social exclusion [M_social exclusion_ = 5.77, SD = 1.24 vs. M_social inclusion_ = 1.89, SD = 1.33; *F*_(1,498)_ = 1141.00, *p* < 0.001], indicating the success of the social exclusion manipulation.

#### Self-Construal

Since independent and interdependent self-construction measures were used to assess different self-construction groups, participants’ self-construction scores represented the level of two different types of construction. To make the level of two constructions comparable, the score of interdependent self was reversely coded. The results of single-factor ANOVA showed that the independent construction score of subjects in the independent self-control group (M_independent self_ = 5.36, SD = 0.80) was significantly higher than that of those in the interdependent-self group [M_interdependent self_ = 2.68, SD = 0.75; *F*_(1,498)_ = 1490.50, *p* < 0.001], suggesting the self-construal control to the subjects was successful.

#### Color Perception

Color perception was tested by using single-factor repeated measures, and subjects reckoned that the color of the purple apartment (M_purple_ = 5.53, SD = 1.18) was cooler than the orange apartment [M_orange_ = 3.91, SD = 1.62; *F*_(1,499)_ = 263.30, *p* < 0.001, η^2^ = 0.35], proving the subjects could effectively distinguish between warm and cold colors.

#### Confounding Check

Firstly, participants’ distinctiveness scores of the two apartments were used as the intra-group repeated measure factor to perform single-factor repeated measure ANOVA. The results showed that the distinctiveness of the purple apartment (M_purple–distinctiveness_ = 5.25, SD = 1.40) was significantly higher than that of the orange apartment [M_orange–distinctiveness_ = 4.82, SD = 1.49; *F*_(1,499)_ = 22.20, *p* < 0.001], indicating there was perception difference in the distinctiveness of the two apartments. To exclude the influence of this variable, it was used as a concomitant variable in the subsequent analysis. Secondly, social exclusion was used as an independent variable, and the subjects’ emotion during the whole experiment were used as the dependent variable for ANOVA. The results showed that the degree of emotion of the participants in the social inclusion group (M_social inclusion_ = 5.53, SD = 1.05) was significantly higher than that in the social exclusion group [M_social exclusion_ = 5.16, SD = 1.27; *F*_(1,498)_ = 12.22, *p* = 0.001]. A significant difference is seen in the emotions of different social exclusion groups. Therefore, subjects’ emotion was used as a concomitant variable in the subsequent analysis to control its influence on the results of the study.

#### Self-Threat

A multi-factor ANOVA was carried out using social exclusion and self-construction as independent variables, apartment distinctiveness and participants ‘ emotions as concomitant variables, and self-threat as a dependent variable. The results have shown that the main effect margin of social exclusion was significant after the subjects’ emotion and the perception of apartment distinctiveness were controlled [*F*_(1,493)_ = 3.47, *p* = 0.063, η^2^ = 0.01], the main effect of self-construction [*F*_(1,493)_ = 6.62, *p* = 0.010, η^2^ = 0.01] and the interaction effect between social exclusion and self-construction were significant [*F*_(1,493)_ = 20.02, *p* < 0.001, η^2^ = 0.04]. The results of further analysis of planned contrast have shown that when participants were interdependent self-constructed individuals, the sense of self-threat of subjects in the social exclusion group was significantly higher than that of those in the social inclusion group [M_social exclusion–self–threat_ = 3.99, SD = 1.26 vs. M_social inclusion–self–threat_ = 3.18, SD = 1.33; *F*_(1,496)_ = 26.11, *p* < 0.001, η^2^ = 0.05]. On the contrary, when participants were independent self-constructed individuals, there was no obvious difference of the sense of self-threat between subjects in the social exclusion group and the social inclusion group [M_social exclusion–self–threat_ = 3.28, SD = 1.28 vs. M_social inclusion–self–threat_ = 3.37, SD = 1.09; *F*_(1,496)_ = 0.33, *p* = 0.569].

#### Product Purchase Intention

A multi-factor covariance analysis was carried out using social exclusion and self-construction as independent variables, emotion and apartment uniqueness as concomitant variables, and product purchase intention as a dependent variable. The results have shown that after controlling the participants’ emotion and product distinctiveness perception, the main effect of social exclusion was significant [*F*_(1,493)_ = 6.93, *p* = 0.009, η^2^ = 0.01], and the main effect of self-construction was not significant [*F*_(1,493)_ = 1.99, *p* = 0.159], while the interaction effect between social exclusion and self-construction was significant [*F*_(1,493)_ = 4.45, *p* = 0.035, η^2^ = 0.01]. The results of further analysis of planned contrast have shown that when subjects were interdependent self-constructed individuals, subjects in the social exclusion group preferred orange apartment (warm color) to those in the social inclusion group [M_social exclusion–orange preference_ = 3.03, SD = 2.41 vs. M_social inclusion–orange preference_ = 3.96, SD = 1.94; *F*_(1,496)_ = 12.56, *p* < 0.001, η^2^ = 0.03]. On the contrary, when participants were independent self-constructed individuals, no obvious difference of the preference of the two apartments is observed between subjects in the social exclusion group and the social inclusion group [M_social exclusion–orange preference_ = 3.71, SD = 1.92 vs. M_social inclusion– orange preference_ = 3.85, SD = 1.97; *F*_(1,496)_ = 0.27, *p* = 0.604], thus Hypothesis 1 was verified again.

#### Moderation Mediation Analysis

The moderating and mediating role of self-construction in the relationship between social exclusion and preference for warm color products have been further verified. Hayes (2013) Process Model 8 was used for the moderating and mediating analysis and there were 5,000 samples. Social exclusion was used as an independent variable (X; 1 = social inclusion, 0 = social exclusion), self-construction was used as a moderating variable (W; 1 = independent self, 0 = interdependent self), the mediating variable (M) was self-threat, and the dependent variable (Y; The smaller the Y value, the more preference for warm color products) was the purchase intention. The results have shown that the type of self-construction played a moderating and mediating role in the influence of social exclusion on product purchase intention (β = –0.128, SE = 0.077, 95%CI = [–0.309, –0.006]). As expected, in the interdependent self-constructed group, self-threat played a mediating role in the influence of social exclusion on product purchase intention. The mediating effect was 0.115 (β = 0.115, SE = 0.065, 95%CI = [0.006, 0.258]). By contrast, in the independent self-constructed group, the mediating effect of self-threat on the influence of social exclusion on product purchase intention was not significant (β = -0.013, *SE* = 0.026, 95%CI = [–0.088, 0.023]). Thus, Hypothesis 3 in the study was verified.

## General Discussion

### Conclusion

Although there are already research on investigating how consumption types can eliminate the negative effects of social exclusion, the mitigation effects of such behavior remains to be explored. This study directly explores the impact of social exclusion on consumers’ color preference, and verifies its internal impact mechanism: self-threat. The results have greatly enhanced the significance of vision in social exclusion and consumption situations. Furthermore, the study verifies the intermediary role of self-threat between social exclusion and color preference (warm vs. cold) through empirical research, and puts forward a new regulatory variable self-construal. Thus, it enriches the theoretical research of social exclusion and the theoretical significance of sensory influence. The main conclusions of this study are as follows: (1) compared with socially included consumers, excluded consumers are more likely to choose warm color products; (2) Self-threat mediates the effect of social exclusion on consumers’ preference for warm (vs. cold) products; (3) Self-construal plays a moderator role in main effect. Specifically, compared to independent self-construal, interdependent self-construal will express greater preference for warm colors.

### Theoretical Contribution

Social exclusion widely exist in daily life, and can lead to impacts on individual’s behaviors. This study discusses the impact of social exclusion on consumer behavior from the perspective of sensory marketing, which has positive theoretical significance. The existing social exclusion literature mostly focus on prosocial behavior, antisocial behavior, conspicuous consumption, unique behavior (Twenge et al., 2001; Maner et al., 2007; Lee and Shrum, 2012; Wan et al., 2014). Although there’s some research on how different consumption types can eliminate the negative effects of social exclusion, the mitigation of such effects by color consumption preference remains to be explored. It introduces a new research perspective which not only expands the existing theories, but also provides new strategies to deal with the negative effects of social exclusion.

On the other hand, this paper supplements and expands the visual marketing in sensory marketing, although predecessors have proposed that hue can also affect the perception of color. Hue will also influence the perception of color. Color itself does not have the characteristics of coldness and warmth, but the association caused by it will make people feel cold or warm. Cold or warm colors will also bring changes in position perception. Warm colors with long wavelengths such as red and orange will form an inner image on retina, resulting in a sense of advancement, while cold colors with short wavelengths such as blue and blue-green will form an outer image, resulting in a sense of retreat. People will feel that time passes faster when they are in an environment with cold colors. While in that with warm colors, it will be just the opposite. Because warm colors symbolize the sun and fire, which can make people feel warm psychologically, while cold colors symbolize water, ice and sky, making people feel cold in their hearts (Elliot and Maier, 2014). However, there is no direct concern about the impact of social exclusion on consumers’ choice behavior. This study directly explores the impact of social exclusion on consumers’ color preference behavior, and puts forward and verifies its internal impact mechanism: self-threat, which has greatly enhanced the significance of vision in social exclusion and consumption situations. Through empirical research, the study verifies the intermediary role of self-threat between social exclusion and cold and warm color preference, and puts forward a new regulatory variable self-construction, which enriches the theoretical research of social exclusion and the theoretical significance of sensory influence.

### Managerial Implications

The findings of this paper have positive implications and value for mitigating the negative effects of social exclusion. For example, businesses can use warm color posters, advertisements, or other product features in their marketing practices to address service failures caused by social exclusion or to increase the satisfaction and success rate of service remedies. Therefore, in the case of service failure caused by social exclusion, warm-colored products or environment can be provided to compensate for the negative consequences of service failure.

For companies that want to produce or sell new products with warm colors, they can set the target market at the rejected consumers, such as people who failed to find a job, to better understand the psychological needs of customers and provide more satisfactory products and services. Companies can also explore the design of feasible warm-colored products to alleviate the negative emotional and behavioral reactions of socially excluded groups, thus increasing the subjective well-being of this special group of people.

In addition, for individuals, our research reveals that people experienced social exclusion tend to feel self-threat. To eliminate their negative psychological feeling, it is important to create a sense of belonging for them, like communicating with them to show you understand their situation and are ready to provide support, or organizing group activities to help them feel valued and welcomed. For government, they should pay more attention to the social-excluded person and launch projects or activities to tackle social exclusion related issues.

### Limitations and Future Research

This study explored the effects of social exclusion on different color preferences from a visual perspective and analyzed the role of self-threat as a mediating mechanism and the moderating role of self-construction. However, there are still some limitations and shortcomings in this study, and further research is needed in the future.

Firstly, social exclusion is complex and multi-dimensional, involving not only material deprivation but also lack of control over important decisions and feelings of alienation and inferiority. Specifically, it entails aspects ranging from age, sex, race, income, education, migration status, ethnicity, religion, disability, to socioeconomic status, and place of residence. Social-excluded individuals cannot participate in the normal relationships and activities. Different types of social exclusion elicit different behavioral responses from people (Lee and Shrum, 2012). However, this study has not distinguished between different types of social exclusion. Future research should focus on the effect of certain types of social exclusion on consumers’ behavior.

Secondly, the dependent variable in this study’s hypothesis test is the willingness to purchase warm and cold products, and the effect of social exclusion on consumers’ actual consumption behavior is not tested. Therefore, the study of this paper would be more valuable if it was transformed from an experimental study to a field research study.

Thirdly, this paper examines how personal self-threatening perceptions influence consumers’ responses to the environmental cue of color. Future research could also examine whether personal self-threat affects people’s responses to other environmental cues, while this paper explores the effect of environmental color on the evaluation of a new product, but whether the color of the new product itself also affects people’s evaluation of that product. Based on intuition, ambient color is different from the product’s own color, and ambient color is less noticeable, but people use product color to determine product preference. Thus, the mechanisms by which product color and environmental color influence the evaluation of a new product may differ, and future research could examine the specific differences. Finally, the independent variable “social exclusion” was controlled in different situations in the three major experiments of the study. Meanwhile, the dependent variables were measured and tested in different categories of products. This strategy is to empower the study with more practical significance, and at the same time enable the results of the study to be popularized and applied in more categories of products. To check whether this strategy can verify the research conclusion again, the measurement method of dependent variables can be fixed and the control situation of ‘social exclusion’ can be changed at the same time.

## Data Availability Statement

The original contributions presented in the study are included in the article/supplementary material, further inquiries can be directed to the corresponding author.

## Ethics Statement

Ethical review and approval was not required for the study on human participants in accordance with the local legislation and institutional requirements. Written informed consent to participate in this study was provided by the participants’ legal guardian/next of kin.

## Author Contributions

LZ and SW contributed to conception and design of the study. LZ organized the database and wrote the draft of the manuscript. LZ and SD performed the statistical analysis. SW and SD revised the manuscript. All authors contributed to manuscript revision, read, and approved the submitted version.

## Conflict of Interest

The authors declare that the research was conducted in the absence of any commercial or financial relationships that could be construed as a potential conflict of interest.

## Publisher’s Note

All claims expressed in this article are solely those of the authors and do not necessarily represent those of their affiliated organizations, or those of the publisher, the editors and the reviewers. Any product that may be evaluated in this article, or claim that may be made by its manufacturer, is not guaranteed or endorsed by the publisher.
